# Intranasal delivery of replicating mRNA encoding neutralizing antibody against SARS-CoV-2 infection in mice

**DOI:** 10.1038/s41392-021-00783-1

**Published:** 2021-10-25

**Authors:** Jia-Qi Li, Zhe-Rui Zhang, Hong-Qing Zhang, Ya-Nan Zhang, Xiang-Yue Zeng, Qiu-Yan Zhang, Cheng-Lin Deng, Xiao-Dan Li, Bo Zhang, Han-Qing Ye

**Affiliations:** 1 Key Laboratory of Special Pathogens and Biosafety, Wuhan Institute of Virology, Center for Biosafety Mega-Science, Chinese Academy of Sciences, Wuhan, China; 2 University of Chinese Academy of Sciences, 100049 Beijing, China; 3 School of Medicine, Hunan Normal University, 410081 Changsha, China

**Keywords:** Microbiology, Molecular biology

## Abstract

The lung is the prophylaxis target against SARS-CoV-2 infection, and neutralizing antibodies are a leading class of biological products against various infectious viral pathogen. In this study, we develop a safe and cost-effective platform to express neutralizing antibody in the lung with replicating mRNA basing on alphavirus replicon particle (VRP) delivery system, to prevent SARS-CoV-2 infections. First, a modified VEEV replicon with two subgenomic (sg) promoters was engineered to translate the light and heavy chains of antibody simultaneously, for expression and assembly of neutralizing anti-SARS-CoV-2 antibody CB6. Second, the feasibility and protective efficacy of replicating mRNA against SARS-CoV-2 infection were demonstrated through both in vitro and in vivo assays. The lung target delivery with the help of VRP system resulted in efficiently block SARS-CoV-2 infection with reducing viral titer and less tissue damage in the lung of mice. Overall, our data suggests that expressing neutralizing antibodies in the lungs with the help of self-replicating mRNA could potentially be a promising prophylaxis approach against SARS-CoV-2 infection.

## Introduction

Severe acute respiratory syndrome coronavirus 2 (SARS-CoV-2) is responsible for the ongoing pandemic of COVID-19, which has caused the hospitalization and deaths of millions of individuals worldwide (https://covid19.who.int). SARS-CoV-2 typically infects the upper and lower respiratory tracts, causing direct cytotoxic effects and local cytokine-mediated hyperinflammation and acute respiratory distress syndrome (ARDS).^1,2^ The spike (S) protein mediates the entry of the SARS-CoV-2 into the cell and is the most important antigen of the virus.^3^ Multiple vaccines using various vaccine platforms or neutralizing monoclonal antibodies (mAbs) that target the S protein of SARS-CoV-2 have already been explored for clinical usage to prevent SARS-CoV-2 infection.^4^ Vaccines are protective but take time to be effective. The prophylactic mAbs can provide prompt protection against viral infection, but their potential is hampered by the high cost of development and manufacturing.^5^ A rapidly-expressed and cost effective technique is urgently needed to contain SARS-CoV-2 infections during viral epidemics and pandemics.

mRNA technology, that has emerged as a powerful tool for immunotherapy during the last few years, provides an elegant solution to circumvent the problems mentioned above.^5^ Increasing number of mRNA vaccines against infectious diseases and cancers, including COVID-19 vaccine, have been developed over the past decades. However, it is until the last few years that the potential of mRNA as an alternative to the recombinant antibodies has been widely appreciated, not to mention the development of an mRNA-encoded SARS-CoV-2 neutralizing antibody. The mRNA mediated monoclonal antibody expression in vivo have been demonstrated to protect mice against several viral infection.^6–9^ In each case, the heavy and light chains of the mAb were encoded by two separate mRNAs, and the functional mAb could be generated by introducing the mRNAs into cells simultaneously. Efficient mRNA expression requires the mRNA to be packaged into some delivery vehicles with the aid of non-viral lipid nanoparticles (LNP) vector or viral vectors.^10,11^ As a respiratory transmitted pathogen, SARS-CoV-2 primarily infects the lungs. It is more desirable to achieve optimal concentrations of mAb in the lungs for the protection against an infection with SARS-CoV-2.

In this study, we explored the feasibility of using Venezuelan equine encephalitis virus (VEEV) replicon, a replicating mRNA, encoding both the heavy and light chains for antibodies expression, to prevent SARS-CoV-2 respiratory infection by VEEV replicon particles (VRPs) delivery through nasal route. VEEV, a member of the genus *Alphavirus* of the family *Togaviridae*, is a positive-sense, single-stranded RNA virus. The viral replicon RNA, which is a self-replicating replicon mRNA encoding viral replicase proteins (nsP1-nsP4) and expressing the gene of interest in place of viral structural protein genes,^12^ can be packaged into alphavirus replicon particles (VRPs) by supplying the viral structural protein genes in trans.^10–12^ VRPs represent a promising and efficient viral vector for mRNA delivery with a broad range of susceptible host cells and high levels of cytoplasmic protein expression. Our results indicate that nose-to-lung delivery of VRP encoding SARS-CoV-2 specific neutralizing mAb can not only forestall viral infection from the outset, but also achieve adequate concentration of protective mAb in the lungs without needles.

## Results

### Construction of VEEV replicon for neutralizing antibody expression (VEEV-Rep-CB6)

**Fig. 1:**
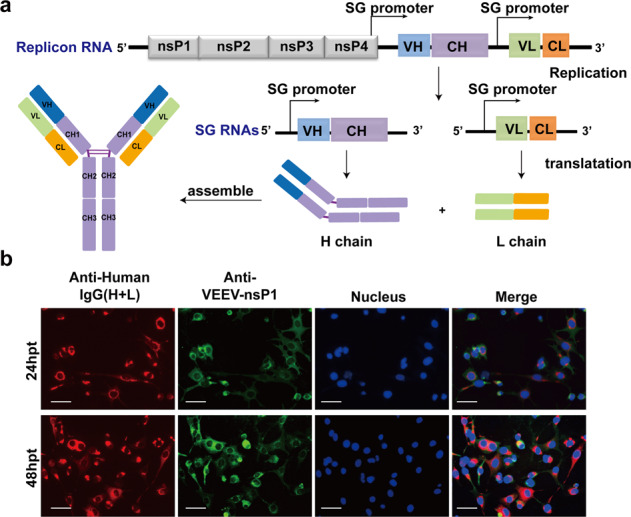
The expression of CB6 mAb using the replicable VEEV replicon as vector. **a** Schematic of the replicable VEEV replicon expressing the heavy (H) and light (L) chains of a human neutralizing anti-SARS-CoV-2 monoclonal antibody CB6. **b** BHK-21 cells were transfected with 5 μg VEEV-rep-CB6 mRNA, and the transfected cells were fixed and stained for the expression of CB6 mAb (red) and VEEV-nsP1 (green) using Alexa Fluor^TM^ 568 conjugated goat anti-human IgG (H + L) and anti-VEEV nsP1 mouse serum, respectively, at 24 and 48 h post-transfection (hpt). Nuclei were stained with DAPI (blue). Scale bars represent 50 μm

To express the heavy and light chains of the neutralizing mAb simultaneously in one VEEV replicon vector, a modified version of VEEV replicon containing two subgenomic (sg) promoters was developed (Fig. 1a). The publicly available sequence of a human monoclonal neutralizing anti-SARS-CoV-2 antibody, named CB6, was used as the candidate sequence of mAb in our study.^13^ The coding sequences of the heavy and light chains of CB6 were under control of two independent sg promoters (Fig. 1a), respectively for neutralizing antibody expression (VEEV-Rep-CB6). BHK-21 cells were transfected with replicon of VEEV-Rep-CB6, and the expression and assembly of CB6 mAb in transfected cells was demonstrated by indirect immunofluorescence assay (IFA) using the goat anti-human IgG (H + L) conjugated with Alexa Fluor^TM^ 568. To detect the replication of the VEEV-Rep-CB6 mRNA, the expression of VEEV non-structural protein nsP1 was detected at different time points post transfection. As expected, strong CB6-specific fluorescence signals were intensely found in transfected cells, and co-localized well with VEEV nsP1 at 24 and 48 h post-transfection (hpt) (Fig. 1b), which indicated the expression of the light and heavy chains of CB6 mAb and the formation of the whole IgG antibody by the replicable VEEV-Rep-CB6 mRNA.

### VEEV replicon particles (VEEV-VRP-CB6) assembly for neutralizing antibody delivery

**Fig. 2:**
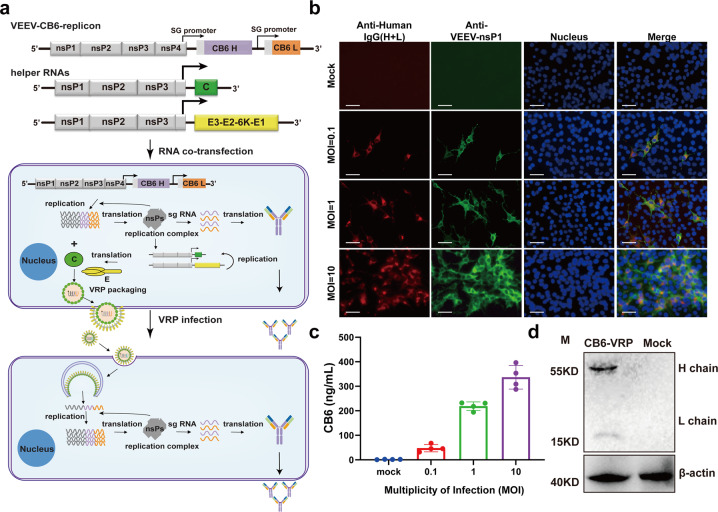
The expression of CB6 with VEEV-VRP delivery in vitro. **a** Schematic representation of the packaging system of VEEV-VRP-CB6 including the VEEV-rep-CB6, and two helper RNAs as described previously,^32^ and the transduction processes of BHK-21 cells with VEEV-VRP-CB6. **b** BHK-21 cells were infected with VEEV-VRP-CB6 at different MOIs (0.1/1/10), the cells were fixed and stained for the expression of CB6 mAb (red) and VEEV-nsP1 (green) using Alexa Fluor^TM^ 568 conjugated goat anti-human IgG (H + L) and anti-VEEV nsP1 mouse serum respectively at 24 h post-infection (hpi). The mock-infected BHK-21 cells were used as a negative control. Nuclei were stained with DAPI (blue). Scale bars represent 50 μm. **c** Quantification of CB6 expression levels by antigen (RBD)-specific ELISA using the supernatants collected at 24 hpi from the VEEV-VRP-CB6 infected BHK cells with different MOIs (0.1/1/10) and mock treated BHK-21 cells. The antibody titers were determined in triplicates. **d** The VEEV-VRP-CB6 infected BHK-21 cells (MOI = 1) and the mock treated cells were lysed with RIPA at 48 hpi and subjected to western blotting using the HRP conjugated goat anti-human IgG (H + L) and the anti-β-actin antibody. The expression of β-actin was used as an internal control

Then, VEEV-Rep-CB6 replicon was packaged into VRP, named VEEV-VRP-CB6, with the help of two helper RNAs encoding VEEV capsid and envelope proteins respectively (Fig. 2a). The resulting VEEV-VRP-CB6 were next used for in vitro delivery of the CB6 neutralizing mAb. Vero-E6 cells were first infected with VEEV-VRP-CB6 at MOIs of 0.1, 1, and 10, respectively, to examine the expression of the neutralizing antibody. As shown in Fig. 2b, increasing IFA positive cells of CB6 mAb and VEEV nsP1 were observed within VRP-infected cells at 24 h post-infection (hpi) with the increase of VRP infection amounts, whereas no expression of CB6 and nsP1 was detected in the uninfected cells. Similarly, increasing levels of antibody titers were detected in the supernatants of VRP-infected cells through enzyme-linked immunosorbent assay (ELISA) using purified RBD domain of spike protein of SRAS-CoV-2 as antigen (Fig. 2c), implying the assembly and secretion of CB6 antibody. Additionally, the integrity of CB6 mAb was confirmed by Western blotting (WB) using goat anti human IgG(H + L) antibody conjugated with HRP (Fig. 2d).

### Functional in vitro validation of VEEV-VRP-CB6 against SARS-CoV-2 infection in cell culture

**Fig. 3:**
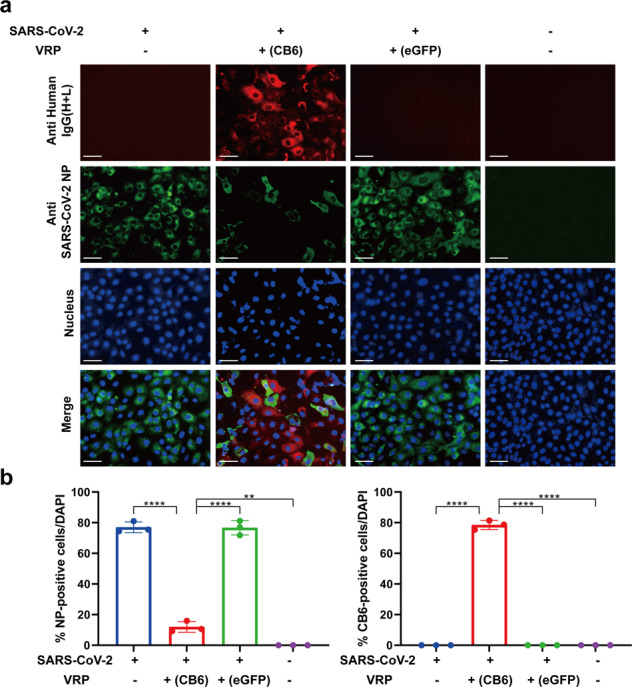
The VEEV-VRP-CB6 treatment could protect cells against SASR-CoV-2 infection. **a** Vero-E6 cells were treated with VEEV-VRP-CB6 or VEEV-VRP-eGFP at the MOI of 10 or DMEM. After 24 h, the transduced cells were infected with SARS-CoV-2 at an MOI of 0.01, and 24 h later, the cells were fixed and stained for SARS-CoV-2 NP protein (green) and CB6 mAb (red), respectively. Nuclei were stained with DAPI (blue). Scale bars represent 50 μm. **b** Percentages of SARS-CoV-2 NP positive cells and CB6 mAb positive cells in each group of cells. The ratios of SARS-CoV-2 NP or CB6 mAb positive cells were calculated by division with cell numbers stained with DAPI from three microscopy images which were captured from different view of each sample, and the error bar represent standard deviations

After demonstrating the ability of VEEV-VRP-CB6 to deliver neutralizing antibody expression, we then evaluated the efficacy of VEEV-VRP-CB6 to block SARS-CoV-2 infection (Fig. 3). Vero-E6 cells were first infected with 10^6^ IU VEEV-VRP-CB6 at 24 h prior to SARS-CoV-2 (WIV04)^14^ infection, and SARS-CoV-2 infected cells which were not treated with VEEV-VRP-CB6 were used as control. The efficacy of SARS-CoV-2 infection was evaluated by measuring the expression of the NP antigen in the cells at 24 h after virus infection. Compared with the Vero-E6 cells without VRP treatment, in which approximately 80% were positive for SARS-CoV-2 NP protein, much less NP-positive cells were observed in Vero-E6 cells transduced with VEEV-VRP-CB6 (Fig. 3). To ensure that the inhibition of SARS-CoV-2 is due to CB6 neutralizing antibody expression rather than non-specific interferences or other effects of the replicon/VRP, we prepared a control VRP using VEEV replicon expressing eGFP (VEEV-VRP-eGFP). As shown in Fig. 3a, the treatment of VEEV-VRP-eGFP could not block SARS-CoV-2 infection, although VEEV-eGFP replicon were efficiently delivered into Vero cells. Our results showed that VEEV-VRP-CB6 could efficiently deliver the CB6 mAb expression and effectively block SARS-CoV-2 infection in cell culture.

### The efficient delivery of antibody expression with VEEV-VRP-CB6 in the lung of mice

**Fig. 4:**
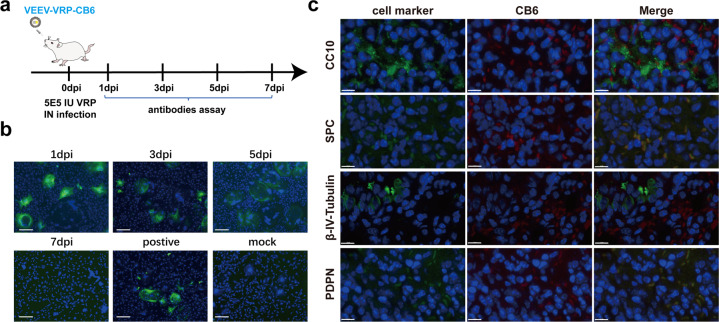
The in vivo expression of CB6 with VEEV-VRP-CB6 delivery. **a** Outline of the experimental design of the expression evaluation of VEEV-VRP-CB6 in BALB/c mice. **b** BALB/c mice received intranasal administration of 5 × 10^5^ IU VEEV-VRP-CB6 were euthanized and the lungs were collected at 1, 3, 5, and 7 dpi. The lung tissue homogenates were used as the primary antibodies to test the Vero E6 cells expressing SARS-CoV-2 S protein with IFA assay, the purified CB6 antibody was used as positive control. Scale bars represent 50 μm. **c** The lung tissues collected at 3 days after the administration of VEEV-VRP-CB6 were subjected to IFA analysis to stain for the expression of CB6 mAb using Alexa Fluor^TM^ 568 conjugated goat anti-human IgG (H + L) and different cellular marker proteins (PDPN/SPC/CC10/β-IV-tubulin), respectively. Scale bars represent 10 μm. (*n* = 2 mice per group)

We then examined the delivery ability of VEEV-VRP-CB6 in vivo for neutralizing antibody expression in the respiratory tract of mouse. BALB/c mice around six to eight weeks-old were first administered with 5 × 10^5^ IU VEEV-VRP-CB6 through intranasal route. The transduced mice were sacrificed and the lung lysates were prepared at 1, 3, 5, and 7 days post VRP infection. To test the specific CB6 mAb expression, we used the lung lysates as primary antibody to detect the Vero E6 cells which were transfected with the SARS-CoV-2 spike protein expression vector. As shown in Fig. 4b, the positive cells were found when the lung lysates collected at 1, 3, and 5 days after VRP infection were used as the first antibody, suggesting that the CB6 antibody could be stably maintained in the lung for at least 5 days.

To further identify the cells types in the lungs of mice transduced with VEEV-VRP-CB6 for neutralizing antibody expression, the lung sections were analyzed for specific lung epithelium cellular makers and CB6 mAb simultaneously through immunofluorescence staining (Fig. 4c). It was found that CB6 mAbs were predominantly expressed within alveolar type I cells (PDPN positive), alveolar type II cells (SPC positive), and club cells (CC10 positive), whose specific markers colocalized with CB6 mAbs, and over 70% of these cells expressed the CB6 antibody. However, CB6 mAb was not detected in ciliated cells (β-IV-tubulin positive). Overall, our results demonstrated that VEEV-VRP-CB6 could efficiently deliver neutralizing antibody expression in most major cell types within the lung of mice.

### Protection efficacy of VEEV-VRP-CB6 against SARS-CoV-2 infection in vivo

**Fig. 5:**
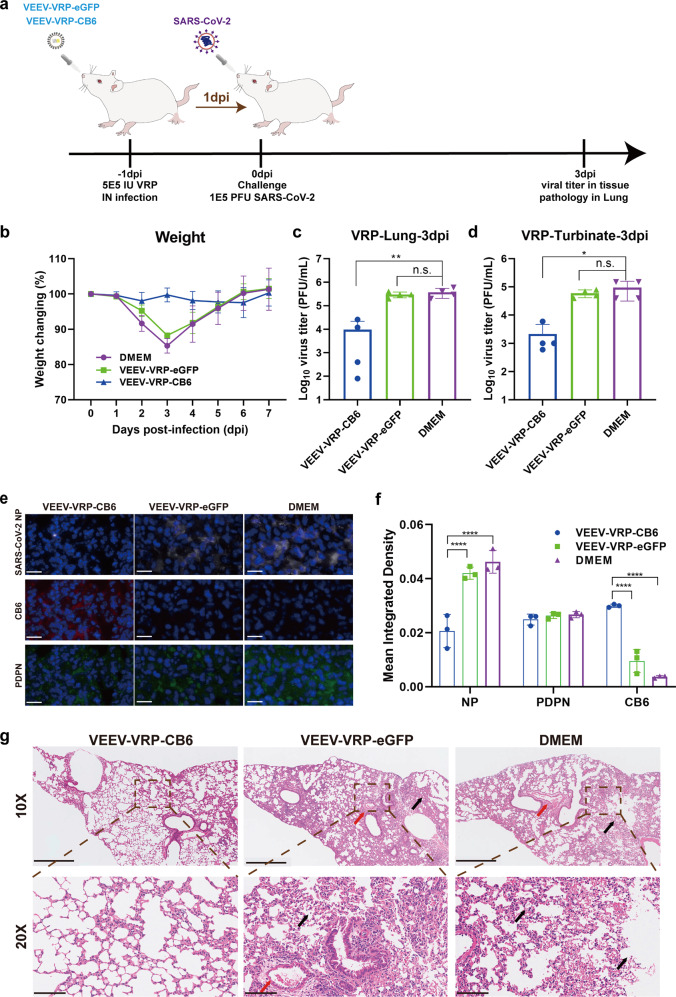
The VEEV-VRP-CB6 infection could protect mice against SARS-CoV-2 challenge. **a** The outline of the experimental design of the in vivo protection evaluation of VEEV-VRP-CB6. Eight-to-ten weeks old BALB/c mice were intranasally administered with 5 × 10^5^ IU VEEV-VRP-CB6, VEEV-VRP-eGFP or DMEM per mouse in a total volume of 50 μL after anesthetization with avertin (250 mg/kg). Twenty-four hours later, the mice from different groups were intranasally challenged with 10^5^ PFU mouse-adapted SARS-CoV-2 strain MP7 in a total volume of 50 μL DMEM, and the body weight changes were recorded daily for 7 days after SARS-CoV-2 infection, the immunofluorescence and pathological analysis of the lung samples collected at 3 days after SARS-CoV-2 infection were performed. **b** Weight changes during 7-day observation period after infection. (*n* = 3 mice per group). **c** Viral titers of lung samples at 3 dpi after SARS-CoV-2 infection (*n* = 4 mice per group). *p = 0.0070 was determined by two-tailed *t*-test. **d** Viral titers of nasal turbinates at 3 dpi after SARS-CoV-2 infection (*n* = 4 mice per group). *p = 0.0379 was determined by two-tailed *t*-test. **e** Immunofluorescence assay of lung samples at 3 dpi after SARS-CoV-2 infection. Tissue sections were stained for the SARS-CoV-2 NP (white), CB6 antibody (red), PDPN cell marker protein (green), and nucleus (blue), respectively. Scale bar represents 20 μm (*n* = 2 mice per group). **f** The fluorescence intensity of the SARS-CoV-2 NP (white), CB6 antibody (red), PDPN cell marker protein (green) in three different views of each slide were calculated by Image J software. *p < 0.0001 was determined by two-tailed *t*-test. **g** Pathological analysis of lung samples at 3 dpi after SARS-CoV-2 infection. Scale bar represents 500 μm and 100 μm (*n* = 2 mice per group), respectively. Inflammatory cell infiltration (black arrow) and focal infiltration of inflammatory cells (red arrow) were indicated

Next, the protection efficacy of VEEV-VRP-CB6 was evaluated in BALB/c mice using the mouse-adapted SARS-CoV-2 strain (MP7), which was selected by our group through serial passage of SARS-CoV-2 in the lungs of old BALB/c mice that could replicate efficiently in the lung of young mice with decreased body weight from day 1 to day 3 of post-infection.^15^ Groups of 8–10 week old BALB/c mice were first inoculated with 5 × 10^5^ IU VEEV-VRP-CB6 or 5 × 10^5^ IU VEEV-VRP-eGFP or DMEM in a volume of 50 μL through intranasal route. After 24 h, the mice from different groups were administered intranasally with 10^5^ PFU MP7, followed by monitoring daily for clinical symptoms and body weight changes (Fig. 5a). No obvious clinical symptoms and body weight loss were observed in the mice transduced with VEEV-VRP-CB6 in contrast to apparent disease manifestations and remarkable reductions in body weight observed in the mice administered with DMEM or VEEV-VRP-eGFP (Fig. 5b). The viral titers in the lungs of mice at 3 days after SARS-CoV-2 infection were measured through plaque assay. Much lower levels of viral titers were found in VEEV-VRP-CB6-treated mice in comparison with DMEM or VEEV-VRP-eGFP treated mice (Fig. 5c). Similarly, lower viral titers were also observed in the nasal turbinate of VEEV-VRP-CB6 transduced mice compared with the mice from other two groups (Fig. 5d). Furthermore, the immunofluorescence assay was performed to analyze the expression of SARS-CoV-2 NP, CB6 mAb, and PDPN (cellular marker of alveolar epithelial type I cells) in the lungs of mice from each group. The fluorescent signals of the three proteins were quantified. As shown in Fig. 5e, f, PDPN was extensively expressed in the lungs of mice of each group, and in the VEEV-VRP-CB6 treated mice, the CB6 antibody was specifically expressed, and much less NP antigen of SARS-CoV-2 was detected compared with the DMEM or VEEV-VRP-eGFP treated mice. Moreover, histopathological analysis of lung sections through hematoxylin-eosin (H&E) staining further demonstrated that much less lesions were visible in the mice treated with VEEV-VRP-CB6 in contrast to developed typical interstitial pneumonia with alveoli atrophy, and large number of inflammatory cell infiltration around blood vessels and the bronchus displayed in VEEV-VRP-eGFP or DMEM-treated mice (Fig. 5g). Overall, our results indicate that such nose-to-lung delivery of VRP encoding SARS-CoV-2 specific neutralizing mAb could forestall viral infection from the outset.

## Discussion

The mAbs are safe pharmaceutical products with the potential for a near-immediate onset of action against prevailing and emerging infectious diseases with potent therapeutic and prophylactic efficacy.^9,16,17^ Their potential is limited by the complexity and high cost of manufacture. The development of mRNA-based antibody platform with the advances in mRNA and formulation technologies offers alternative cost-effective strategy for in vivo antibody expression over massive antibody proteins production and purification. The feasibility of using mRNA to encode antibodies has been demonstrated for the treatment of viruses, toxins and tumors.^6–9,18^ In general, the coding sequences for heavy and light chains of the mAbs are encoded by two separate mRNAs, which are formulated into lipid nanoparticles (LNPs) with ionizable lipids and lipid-like materials for in vivo delivery. To improve the folding and assembly of the whole IgG, it is necessary to optimize the light-to-heavy-chain ratio due to the different lengths and expression rates of the two chains. In this study, using the self-replicating VEEV replicon, we developed a single mRNA vector that expresses the heavy and light chains of CB6 mAb simultaneously (VEEV-rep-CB6), in which the coding sequences of heavy and light chains were under control of two identical subgenomic promoters with the expectation of their equal expression in cells. However, the western blot assay showed that the expression levels of the two chains were different (Fig. 2d). A possible explanation for this phenomenon is the competition between promoters when they are closely spaced, whereby the transcription is more efficient at the stronger promoter, leading to higher expression of the controlled protein.^19,20^ It has been reported that the length and location of the subgenomic promoter affect the expression level of the heterologous protein from the alphavirus replicon vectors.^19,21^ Therefore, the duplicated SG promoter in VEEV-rep-CB6 can be further optimized to increase the expression level of the light chain and achieve higher production of the CB6 mAb.

Currently, most of the mRNAs encoding antibodies are mainly delivered into cells by their encapsulation into LNPs.^6–9^ Although the LNP system is a promising vehicle for mRNA intracellular delivery, its clinical translation is restricted by safety concerns due to the scarcity of clinical data on safety evaluation of LNPs. In terms of viral vector mediated antibody gene delivery strategies, the recombinant adeno-associated virus (AAV) and adenovirus (AdV) platforms have been applied in the prevention of HIV and influenza viruses infections.^22–24^ AAV is non-pathogenic in humans, and can maintain long-term transgene expression in both dividing and non-dividing cells. Different AAV vectors have unique tissue tropisms such as liver, lung, and muscle based on their surface capsid.^25^ These features make the AAV vectors a leading choice for gene delivery studies at present. However, AAV vectors take at least 3–4 weeks to reach a steady expression level of the transgene,^26^ and as a DNA virus, the risk of integration raises a safety concern of their clinical application. The adenovirus vector-mediated antibody delivery has been shown to be rapid and relatively short-term, with antibody detectable within 1–14 days after transduction.^24^ As natural adenovirus infections are common in human population, the widespread pre-existing immunity may hinder the clinical use of the adenovirus vectors. Here, we demonstrated the feasibility of using the alphavirus VRP platform for in vivo antibody delivery. Compared to the DNA virus vectors, the alphavirus VRP mediated antibody expression is more efficient as the replicon mRNA bypasses the intracellular transcription process and directly translate the antibody after transferring into cells. Furthermore, the replicon mRNA circumvents the risk of genomic integration, increasing the safety profile of this strategy. The alphavirus VRP systems have been extensively used in the studies of gene transduction, cancer therapy and vaccine development, and their efficacies and safety have been well evaluated pre-clinically in different animal models including various rodents and non-human primates.^27^ Several clinical trials of the VRP-based vaccines against infectious diseases or cancers have been conducted, and only mild or moderate adverse events with the patterns of response similar with the placebo groups occurred,^28^ confirming the safety and tolerability of the alphavirus VRP platform, and encouraging the clinical translation of VRP-vectored antibody delivery approach.

As a respiratory-transmitted pathogen, the lung is a critical prophylaxis target for SARS-CoV-2 infection. It is important to impart the maximum lung-protective effects. The targeted delivery of the therapeutics to the organ of interest has the potential to minimize anti-antibody immune responses and systemic toxicity.^8^ Delivery of antibody-encoding mRNA directly to the lung will increase concentration of mAbs in the target organ, and reduce the amounts of drug required to achieve therapeutic levels. Thereby, instead of the routinely used administration routs of mAb, like intravenous (i.v) infusion or intramuscular (i.m) injection, we delivered replicating VEEV replicon RNA expressing SARS-CoV-2 neutralizing mAb through intranasal route. Our results addressed the feasibility of local deliver of the mRNA encoding mAb for protection from SARS-CoV-2 infection in mice model. We show that single intranasal administration of VEEV-VRP-CB6 could cause the competent expression of CB6 mAb in the majority cell types within the lung of mice, and the mAbs could be stably maintained for at least 5 days. On the contrary, in the treatment of pulmonary administration of the purified recombinant mAb, large amounts of mAbs are firstly required, and the antibodies were predominately located within the airway lumen but not the lung parenchyma, and would be rapidly eliminated from the lung in 48 h after the mAbs addition.^29^ The VRP delivery strategy of mAb possibly have obvious advantages in the treatment of respiratory disease and have great potential to be translated to human applications. Additionally, nasal delivery also offers benefit over traditional approaches with ease of administration without needles, which will increase acceptance in both adults and children.^30^

Multiple SARS-CoV-2 variants have emerged and circulated globally throughout the COVID-19 pandemic, posing significant challenges to the effectiveness of the existing vaccines and human monoclonal antibodies. Since the B.1.617.2 (Delta) variant was identified in India in late 2020, it has spread fast to many countries and become the dominant strain worldwide currently. A recent study has demonstrated that the sensitivity of Delta variant to several clinically approved monoclonal antibodies was reduced.^31^ For CB6 antibody, it exerts its neutralizing activity by binding with the RBD of SARS-CoV-2 spike protein and blocking the interaction between RBD and the receptor hACE2.^13^ Compared to the original SARS-CoV-2 strain isolated in Wuhan,^14^ the Delta variant contains two substitutions (L452R and T478K) in the RBD of spike protein.^31^ According to the crystal-structure analysis,^13^ neither L452R nor T478K was located within the binding interface between SARS-CoV-2 RBD and CB6 mAb, which should have no influence on the neutralizing activity of CB6 on Delta strain. Therefore, we suppose that VEEV-VRP-CB6 also can effectively prevent the infection of the SARS-CoV-2 Delta variant, and its neutralizing activity should be evaluated using the live Delta strain virus in the future study.

In summary, our strategy represents a new approach for mRNA-based mAbs expression and also provides alternative effective approach for prevention against other respiratory-transmitted pathogens with the help of VRP delivery system through nasal inoculation.

## Materials and methods

### Cells, viruses, and antibodies

BHK-21 and Vero-E6 cells were cultured in Dulbecco’s modified Eagle’s medium (DMEM) containing 10% fetal bovine serum (FBS), 100 units penicillin mL^−1^ and 100 µg streptomycin mL^−1^ in 5% CO_2_ at 37 °C. SARS-CoV-2 (IVCAS 6.7512) and mouse-adapted SARS-CoV-2 strain (MP7) were propagated in Vero-E6 cells and titrated by single layer plaque assay with standard procedure.^15,32^ Murine polyclonal antiserum against VEEV-nsP1 proteins were generated in our lab. Murine monoclonal antibodies targeting mouse PDPN/SPC/CC10/β-IV-tubulin were purchased from Abcam, UK. Fluorescein isothiocyanate (FITC)-/Horseradish Peroxidase (HRP)-conjugated goat anti-mouse/rabbit/human IgG, and Alexa Fluor 568-anti human IgG were purchased from Proteintech, China.

### Plasmid construction

The cDNA clone of VEEV replicon (pACYC-VEEV-Rep) with the entire deletion of the structural protein genes in the VEEV vaccine strain TC83 was used for CB6 mAb expression. To express the heavy and light chains of mAb simultaneously, a second subgenomic promoter (5′-GGGCCCCTATAACTCTCTACGGCTAACCTGAATGGACTACGACAT-3′) was inserted downstream the original SG promoter into the VEEV replicon vector. The coding sequences of the heavy and light chains of CB6 mAb (GenBank: MT470197.1 and MT470196.1, respectively) were in vitro synthesized from Shenggong, China, and engineered into the VEEV replicon downstream the two independent SG promoter, respectively. The cDNA clones of the two helper RNAs which encode the capsid and envelop proteins (designated as VEEV-del nsP4-C and VEEV-del nsP4-E, respectively) were constructed in our previous study.^32^ All the plasmids were validated by DNA sequencing analysis before the subsequent experiments.

### Production and titration of VRP expressing CB6

The RNAs used for packaging VRP were in vitro transcribed using a mMESSAGE mMACHINE ^TM^ T7 Transcription Kit (Invitrogen) according to the manufacturer’s protocols. To generate the VEEV-VRP-CB6, 8 × 10^6^ BHK-21 cells in 0.8 mL ice-cold PBS were electroporated with 5 μg of each VEEV-rep-CB6, VEEV-del nsP4-C and VEEV-del nsP4-E RNAs in a 0.4 cm cuvette with a GenePulser apparatus (Bio-Rad) at 0.85 kV and 25 μF, pulsing three times at 3 s intervals. The electroporated cells were seeded in a T75 flask and incubated at 37 °C in 5% CO_2_. The supernatants of the cells were harvested at 24 h post transfection. The titer of VRP, which was expressed as infectious units (IU) per mL, was determined by IFA assay. Briefly, the supernatants were 10-fold serially diluted followed to infect BHK-21 cell monolayers in 24-well plates. After 24 h, the infected cells were fixed with cold (−20 °C) 5% acetic acid in methanol, and subjected to immunofluorescence assay using the FITC conjugated goat anti-human IgG antibody.

### Western blotting assay

BHK-21 cells were infected with VEEV-VRP-CB6 at a multiplicity of infection (MOI) of 1. At 48 hpi, the cells were lysed in RIPA buffer (Beyotime). The samples were analyzed by SDS-PAGE and transferred onto PVDF membrane. After being treated with skimmed milk for 2 h, PVDF membrane was incubated with secondary horseradish peroxidase (HRP)-conjugated anti-human IgG. The signals were detected with a chemiluminescence system (ChemiDoc, Bio-Rad) using Pierce ECL Western Blotting Substrate kit.

### Immunofluorescence assay (IFA)

Vero-E6 cells were infected with VEEV-VRP-CB6 or VEEV-VRP-eGFP at an MOI of 1 for 24 h, followed by infection with SARS-CoV-2 (MOI = 0.01). At 24 h after SARS-CoV-2 infection, the cells were fixed with 4% paraformaldehyde and treated with 0.1% Triton-X 100 in PBS for 15 min at room temperature. For the detection of CB6 expression, Alexa Fluor 568-anti human IgG was used as secondary antibody. Viral replication was detected using anti-NP antibody and FITC-conjugated goat anti-rabbit IgG as primary and secondary antibodies, respectively. The nuclei were stained with DAPI. Fluorescence images were obtained with a Nikon confocal microscope.

### Plaque assay

Approximately 1 × 10^5^ Vero-E6 cells per well were seeded into 24-well plates one day before plaque assay. A series of 1:10 dilutions were prepared by mixing 15 μL of virus stock with 135 μL of DMEM containing 2% FBS. Then, 100 μL of each dilution was added to each well of 24-well plates containing confluent Vero-E6 cells. The plates were incubated at 37 °C with 5% CO_2_ for 1 h before the layer of 2% methyl cellulose was added. After 4 days of incubation at 37 °C with 5% CO_2_, the cells were fixed with 3.7% formaldehyde and then stained with 1% crystal violet. Plaque morphology and numbers were recorded after washing the plates with tap water.

### Mice assay

Eight-to-ten weeks old female BALB/c mice were provided by the Animal Center of Wuhan Institute of Virology. All the mice were cared in accordance with the recommendations of National Institutes of Health Guidelines for the Care and Use of Experimental Animals. Viral infections were conducted in an animal biosafety level 3 (ABSL-3) facility at Wuhan Institute of Virology under a protocol approved by the Laboratory Animal Ethics Committee of Wuhan Institute of Virology, Chinese Academy of Sciences (Permit number: WIVA26201701).

To evaluate the efficacy of VEEV-VRP-CB6 in vivo, three groups of female BALB/c mice (*n* = 4 per group) were intranasally infected with 5 × 10^5^ IU VEEV-VRP-CB6, VEEV-VRP-eGFP or DMEM per mouse in a total volume of 50 μL after anesthetization with Avertin (250 mg/kg). Twenty-four hours later, the mice from different groups were intranasally challenged with 10^5^ PFU mouse adapted SARS-CoV-2 strain MP7^15^ in a total volume of 50 μL DMEM. Mice were weighed daily during 7-day observation period. At 3 days after SARS-CoV-2 infection, the mice from each group were euthanized and the lungs and turbinates were collected for viral load determination by plaque assay.

### Histological analysis

Lung samples from mice at 3 dpi were fixed with 4% paraformaldehyde, embedded in paraffin followed by sagittal sections of 4-μm thickness on a microtome, and mounted on APScoated slides. For H&E staining, sagittal sections directly stained with H&E. For detection of SARS-CoV-2 antigen in fixed lungs, indirect immunofluorescence assay (IFA) was conducted. Briefly, the slides were deparaffinized, rehydrated and experienced heatinduced antigen retrieval with EDTA (pH 8.0) in a microwave oven. Then tissues were uniformly covered with 5% BSA for incubation at room temperature for 1 h followed by further incubation with the primary antibody (anti-SARS-nCoV-2 NP protein polyclonal antibody, 1:1000 and anti-cell marker protein PDPN antibody,1:1000) and then washed in PBS. After the slices were slightly dried, tissues were covered with 488s-conjugated goat-anti-rabbit IgG, 617s-conjugated goat-anti-mice IgG and Alexa Fluor 568-anti human IgG at 1:1000 dilution. After washing in PBS, slides were stained with DAPI (Beyotime) at 1:100 dilution. The image information was collected using a Pannoramic MIDI system (3DHISTECH, Budapest) and FV1200 confocal microscopy (Olympus).

### Statistical analysis

All the data were analyzed using GraphPadPrism 8.0.2 software and expressed as mean ± standard deviation (SD). The statistical significance was assigned when *p* values were < 0.05. Student’s *t*-test was used to analyze the differences between two groups, and significant differences between groups were determined using a two-way analysis of variance (ANOVA).

## Data Availability

All data supporting the findings of this study are available within the article or from the corresponding author upon reasonable request.
